# Supplementary material for: Dual functionality of the amyloid protein TasA in Bacillus physiology and fitness on the phylloplane
Source: Nat Commun. 2020 Apr 20;11:1859. doi: 10.1038/s41467-020-15758-z (PMC7171179; doi:10.1038/s41467-020-15758-z)
Supplement: Supplementary file 1 — Supplementary Information [file 41467_2020_15758_MOESM1_ESM.pdf]

Dual functionality of the amyloid protein TasA in *Bacillus* physiology and fitness on the phylloplane

Cámara-Almirón *et al.*

Supplementary material

-Supplementary figures

Supplementary Figure 1

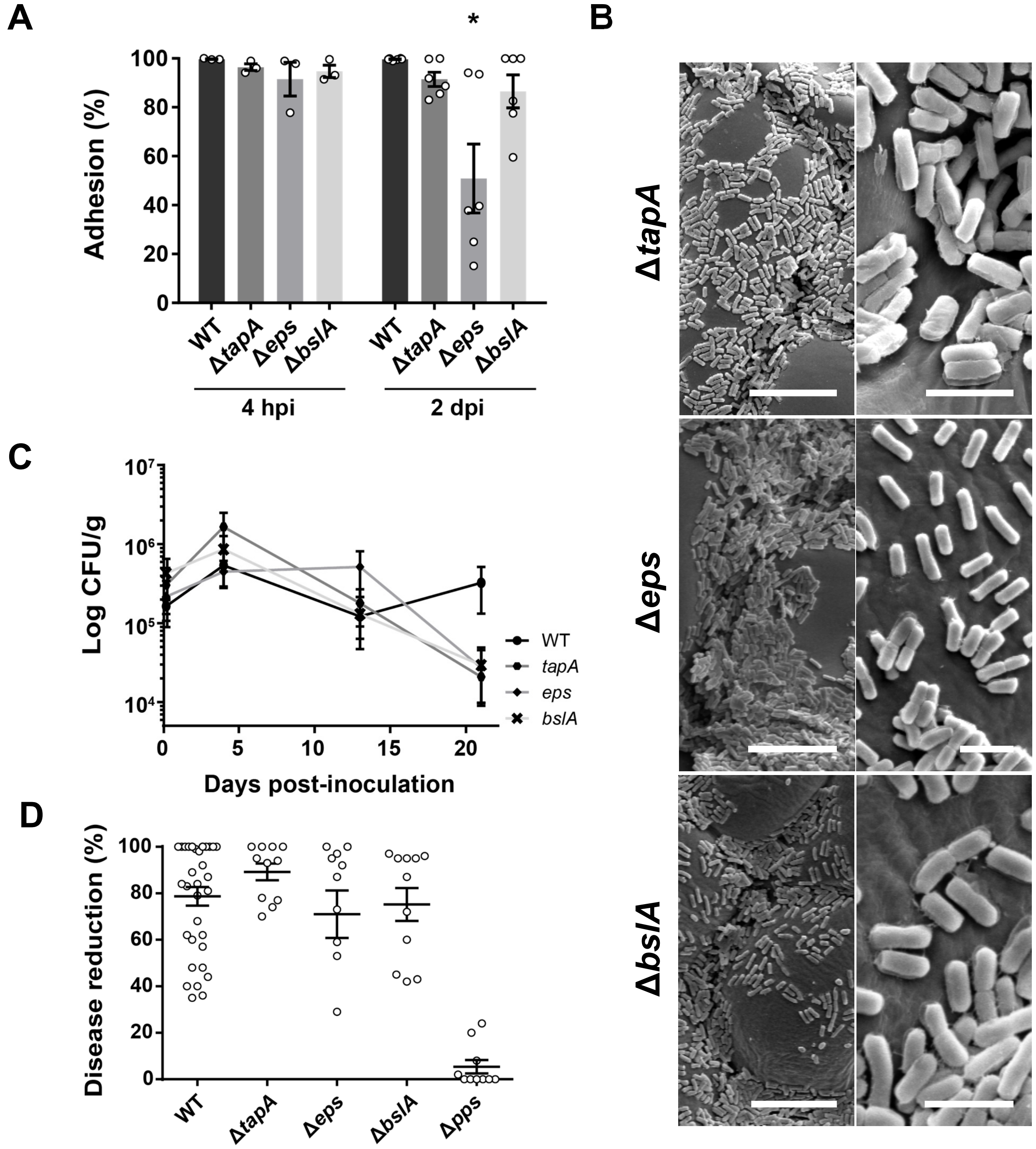

Supplementary Figure 1. Interaction of *Bacillus subtilis* strains with single mutations in ECM components with melon leaves. A) Adhesion of the ECM single mutants to melon leaves 4 h and 2 days post-inoculation (hpi and dpi, respectively). The *tasA* (see Fig. 1) and *eps* mutants were the only matrix single mutant strains whose adhesion to melon leaves was significantly affected 2 days post-inoculation (dpi). At 4 hpi, N = 3 for all the strains. At 2 dpi, N = 6 for all the strains except for the WT strain, where N = 3. N refers to the number independent experiments. In each experiment, 10 leaves were analyzed. Average values are shown. Error bars represent the SEM. Statistical significance was assessed via Kruskal-Wallis test with Dunnet multiple comparisons test (* $p<0.05$). B) The *Δeps*, *ΔbslA* and *ΔtapA* strains showed a colonization pattern in which the cells. were distributed randomly over the leaf surface with no connection between the cells or extracellular material. Scale bars = 12.5 µm (left) or 2.5 µm (right). Experiments have been repeated at least three times with similar results. C) All of the matrix single mutants showed decreased persistence on plant leaves. The first point is taken at 4 hpi. Average values of 5 biological replicates are shown with error bars representing the SEM. D) Strains with *tapA* (N = 11) mutations (i.e., fewer amyloid fibers) showed as much biocontrol activity as that of the WT strain (N = 22). The *eps* (N = 10) and *bslA* (N = 11) mutant strains showed reduced antagonistic activity compared to that of the WT strain. N refers to number of plants analyzed over three independent assays. Three leaves per plant were infected and inoculated. Average values are shown with error bars indicating the SEM. The WT data from figure 1A, 1C and 1D and the Δ*pps* data in figure 1D are from the same experiment as the data displayed in this figure and have been used as controls for the comparison between the WT strain and the ECM single mutant strains. Source data are provided as a Source Data file.

Supplementary Figure 2

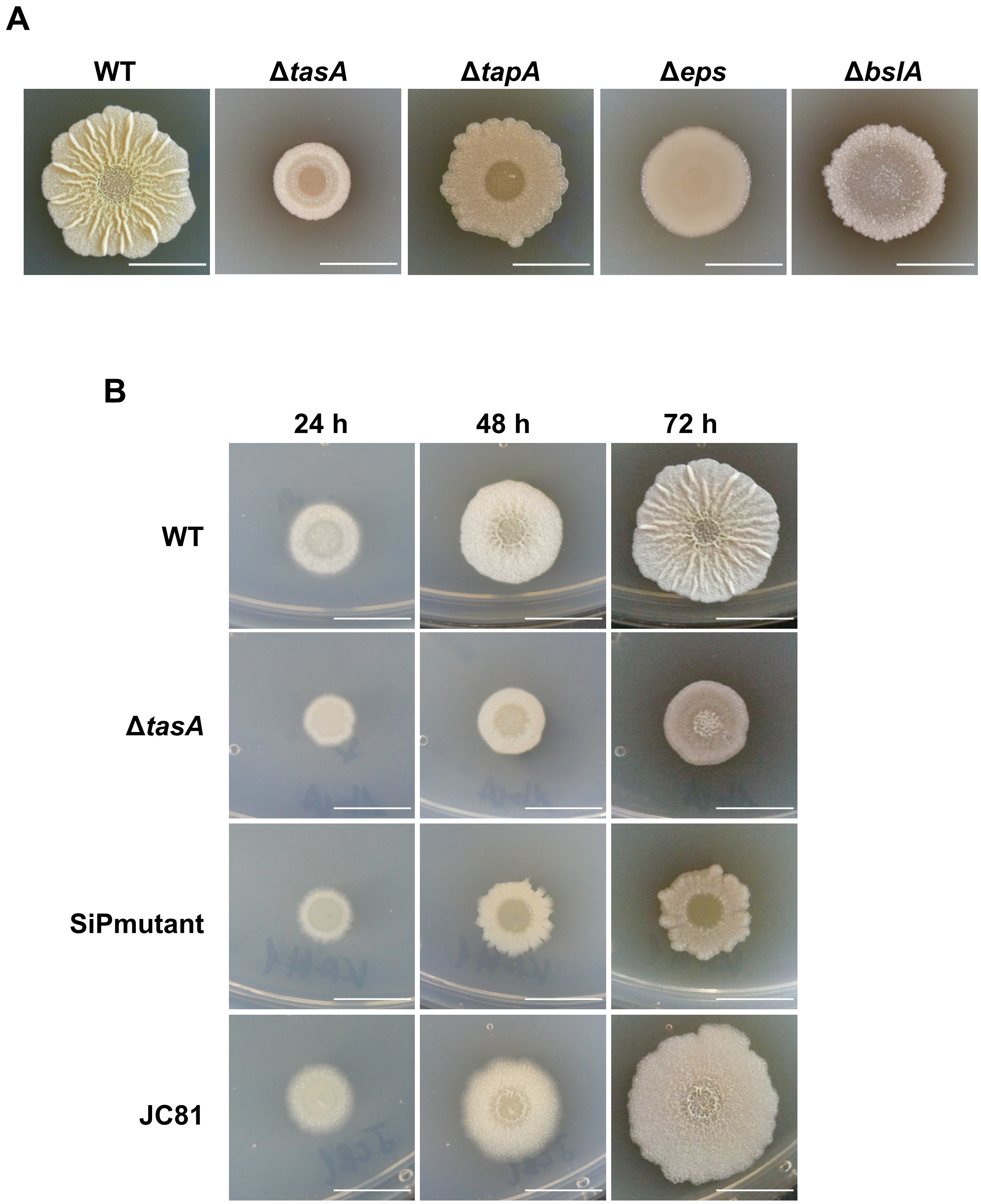

Supplementary Figure 2. Morphology of the *Bacillus subtilis* matrix mutants grown under biofilm-inducing conditions. A) Colony morphologies of the ECM single mutant strains after 72 h of growth on MSgg agar plates. B) Time course of the colony morphologies of the WT, *ΔtasA*, SiPmutant (Lys4Ala, Lys5Ala, Lys6Ala) and JC81 (Lys68Ala, Asp69Ala) strains. Scale bars = 1 cm.

Supplementary Figure 3

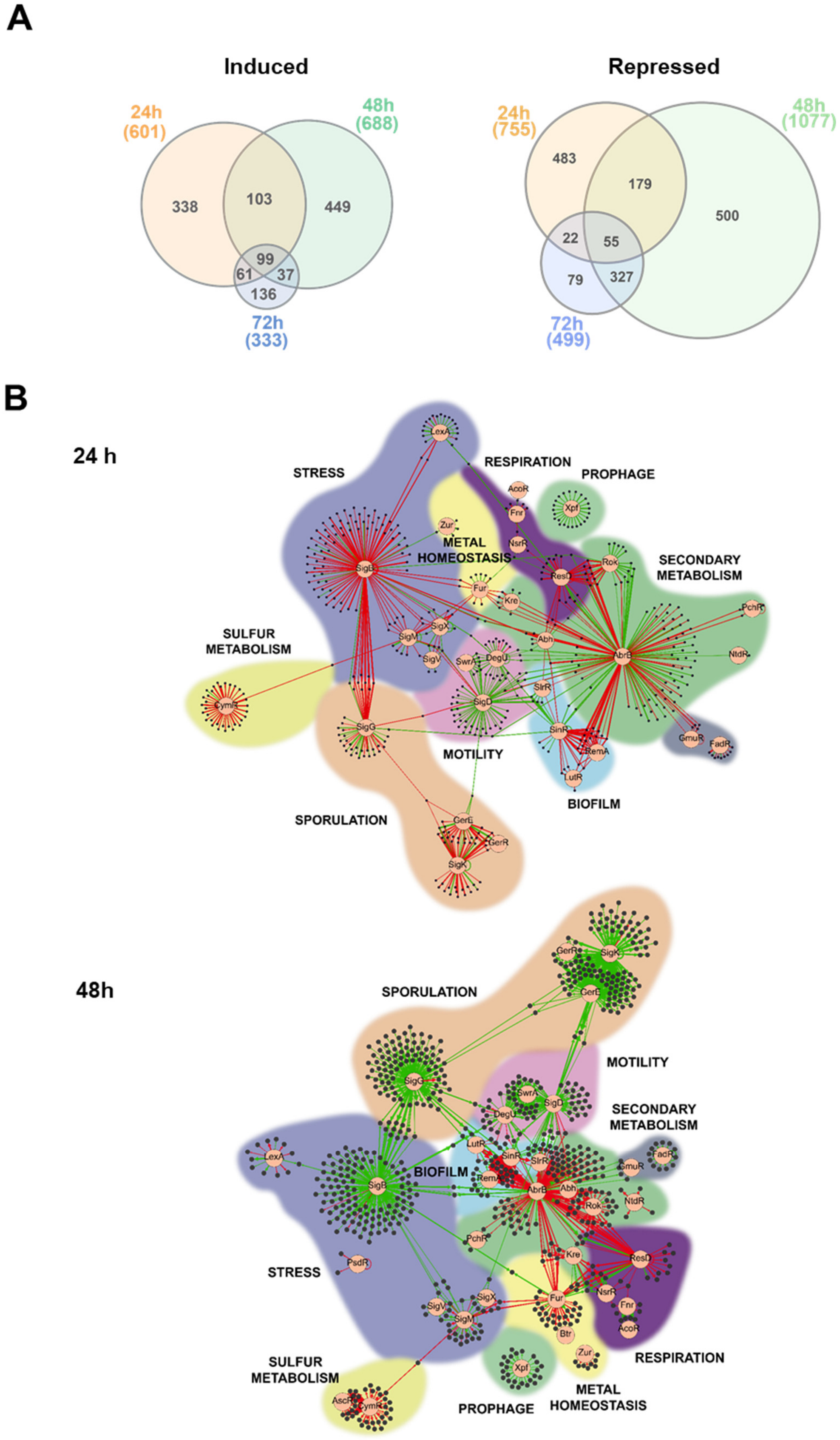

Supplementary Figure 3. Deletion of *tasA* has pleiotropic effects on gene expression. A) Venn diagrams showing the differentially expressed genes at 24 h, 48 h and 72 h in Δ*tasA* compared to the WT. B) Regulation networks of the differentially expressed genes in Δ*tasA* compared to the WT at 24 h (top) and 48 h (bottom). Red indicates induction. Green indicates repression.

Supplementary Figure 4

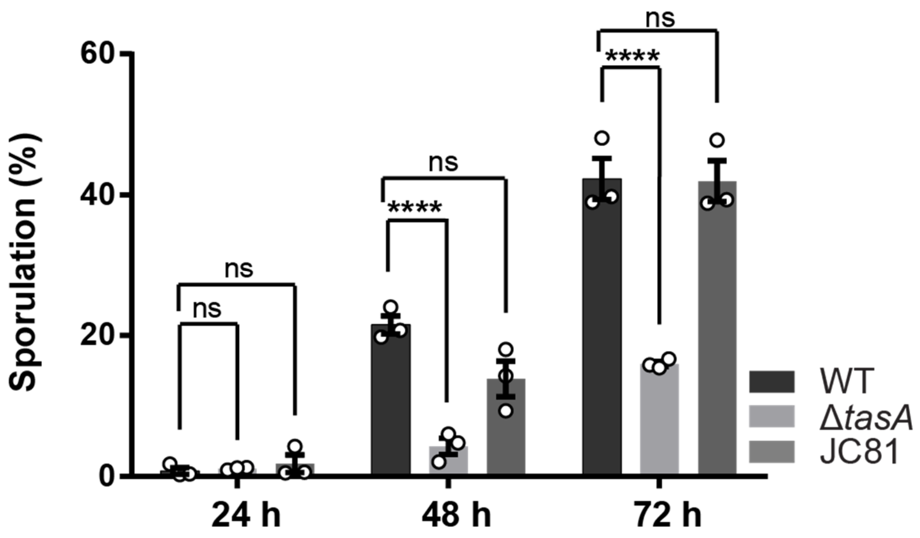

Supplementary Figure 4. The Δ*tasA* strain shows a lower percentage of sporulation compared to those of the WT and JC81 strains. Sporulation rates of the WT, Δ*tasA* and JC81 colonies at 24 h, 48 h and 72 h (N = 3 colonies examined over three independent experiments). The Δ*tasA* colonies showed a sporulation delay typical of ECM mutants with significant differences compared to the WT colonies at 48 h and 72 h. Average values are shown. Error bars represent the SEM. Statistical significance was assessed via two-tailed independent t-tests (**** $p<0.0001$). Source data are provided as a Source Data file.

Supplementary Figure 5

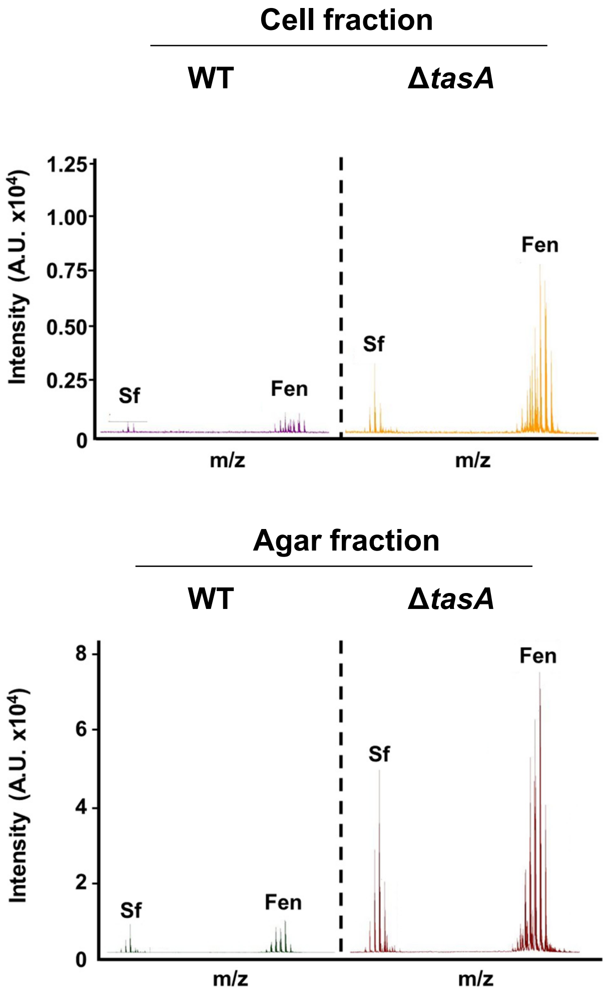

Supplementary Figure 5. Δ*tasA* produced higher relative amounts of fengycin. Mass spectra corresponding to the cells (top) or the agar (bottom) fractions of the WT or Δ*tasA* colonies grown on solid MSgg for 72 h.

Supplementary Figure 6

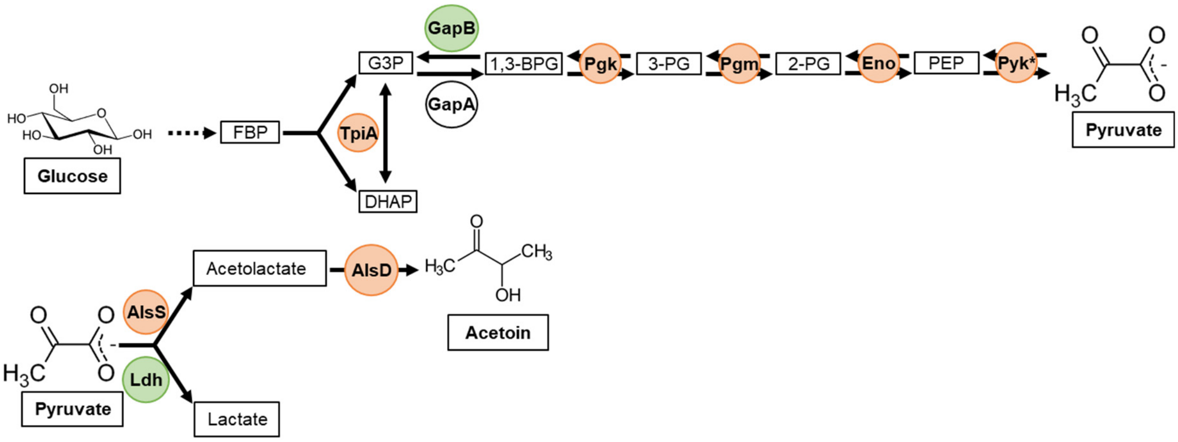

Supplementary Figure 6. Deletion of *tasA* affects genes expression. The diagram shows the pathway that terminates in acetoin synthesis. Some steps of the glycolysis pathway (arrows to the right) are induced in the *ΔtasA* strain, whereas the divergent or gluconeogenetic steps (arrows to the left) are repressed at 72 h. Green indicates repression. Red indicates induction.

Supplementary Figure 7

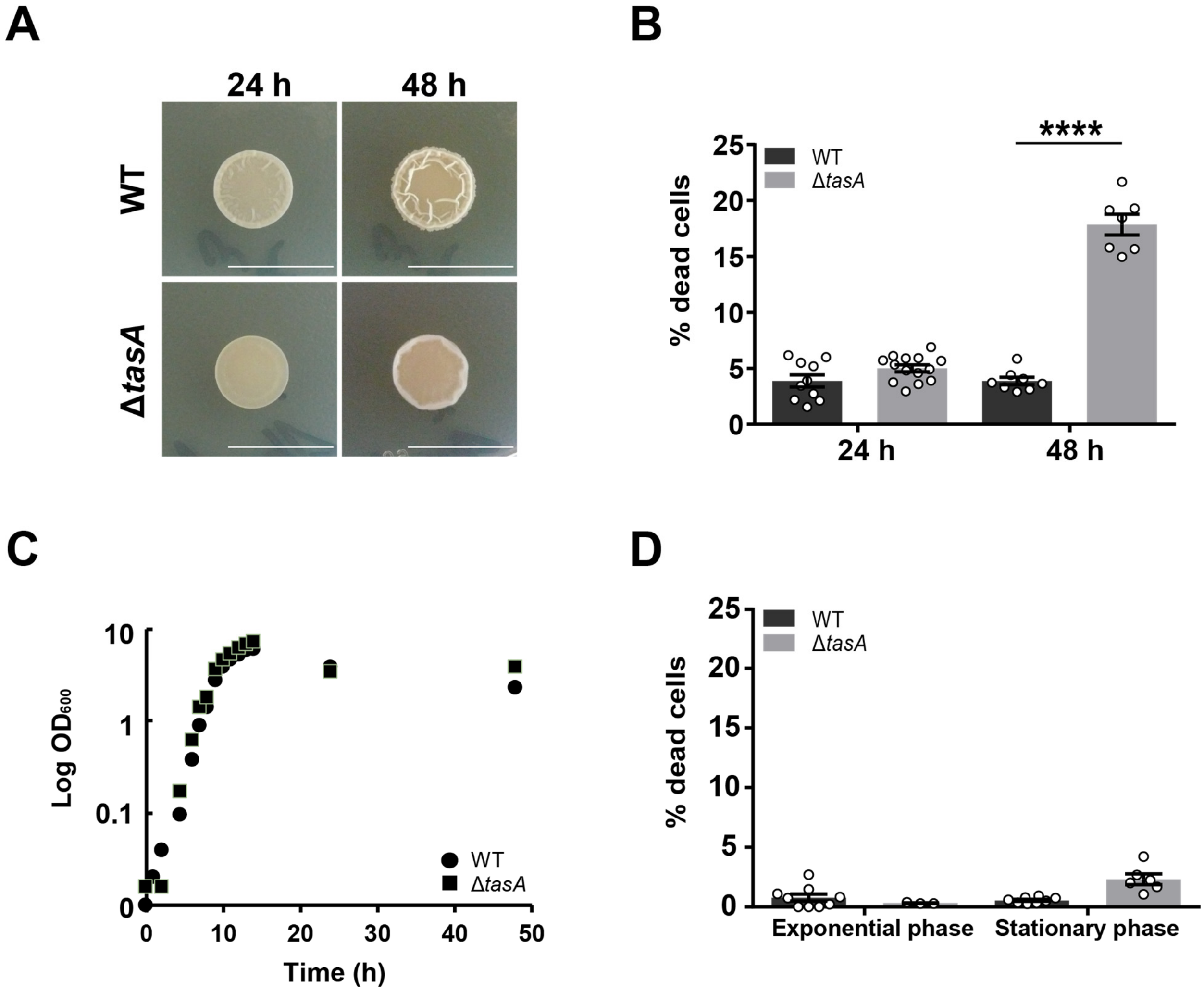

Supplementary Figure 7. The physiological phenotypes observed in Δ*tasA* are independent of media composition. A) Colony morphologies of WT or Δ*tasA* growing in LB plates for 48 h. Scale bars = 1 cm. B) Quantification via CLSM of the proportions of dead cells found in the WT or Δ*tasA* colonies grown on solid LB for 24 (WT N = 11, Δ*tasA* N = 14) or 48 h (WT N = 8, Δ*tasA* N = 7). N represents the number colonies examined over three independent experiments. Average values are shown. Error bars represent the SEM. Statistical significance was assessed via two-tailed independent t-tests at each time-point (**** $p<0.0001$). For each experiment and sample, at least three fields-of-view were measured. C) Growth curves of the WT strain and Δ*tasA* strain in liquid MSgg. No differences were observed in the growth rates of both strains in these experimental conditions. D) Quantification via CLSM of the proportions of dead cells found in the WT or Δ*tasA* liquid MSgg cultures in exponential (WT N = 9, Δ*tasA* N = 3) or stationary phase (WT N = 8, Δ*tasA* N = 6). All samples were measured over three independent experiments. For each experiment and sample, at least three fields-of-view were measured. Average values are shown. Error bars represent the SEM. Source data are provided as a Source Data file.

Supplementary Figure 8

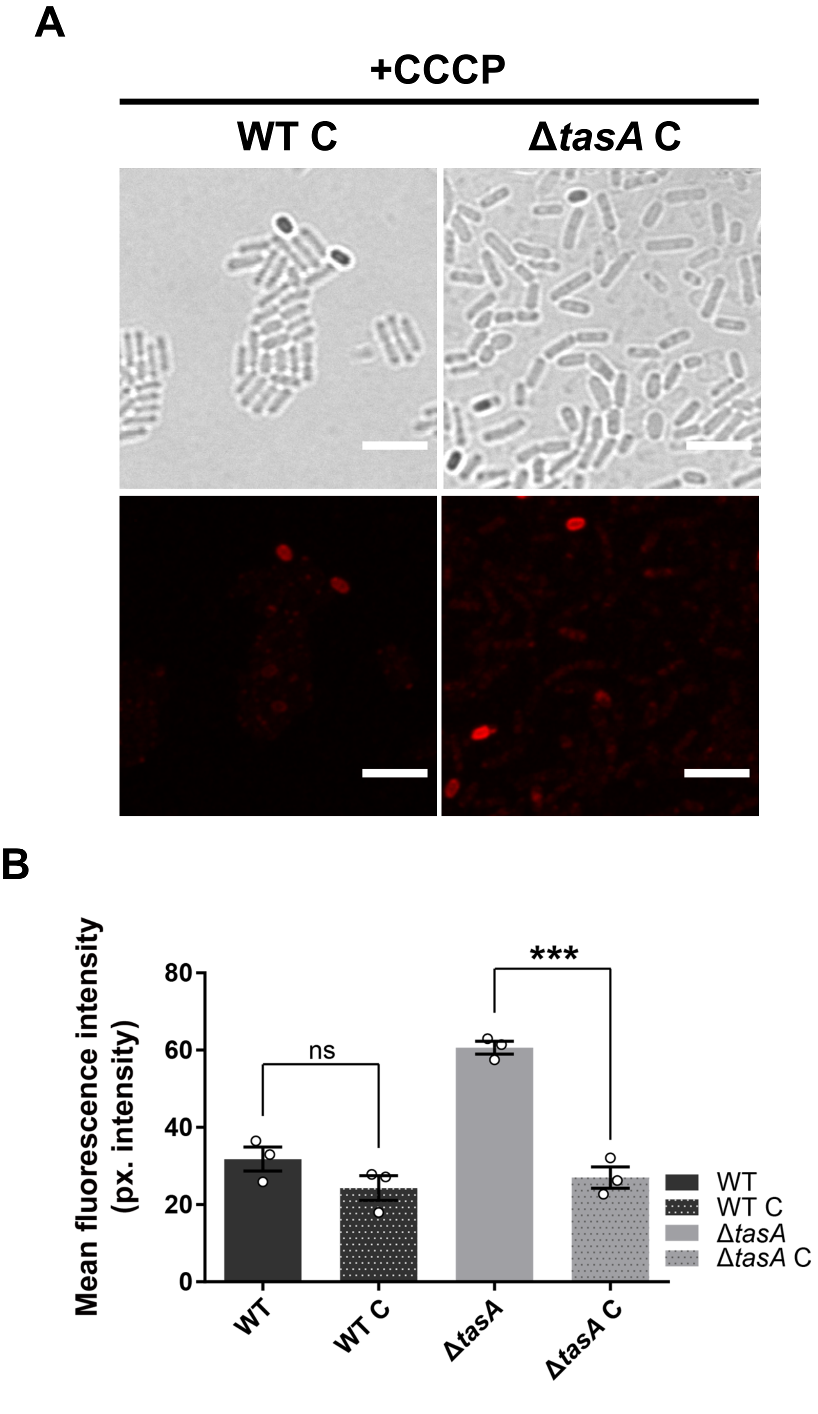

Supplementary Figure 8. Treatment of WT and *ΔtasA* cells with CCCP caused a decrease in membrane potential. A) As a control for the TMRM experiments, CLSM analysis of 72-hour-old WT and *ΔtasA* colonies treated with CCCP, a known ionophore that causes proton gradient disruption and depolarization, was performed. The WT cells showed a natural decay in their membrane potential over time; therefore, at 72 h, CCCP had very little effect (WT C). However, in the *ΔtasA* cells, which showed hyperpolarization at 72 h, CCCP had a major effect on the membrane potential (*ΔtasA* C), as it could depolarize cells to a level comparable to that in WT C. Scale bars = 5 µm. B) Quantification of the TMRM signals in WT C and *ΔtasA* C showed statistically significant differences in Δ*tasA* C at 72 h compared to that of the untreated sample. N = 3 colonies examined over three independent experiments. For each experiment and sample, at least three fields-of-view were measured. Average values are shown. Error bars indicate the SEM. Statistical significance was assessed via two-tailed independent t-tests between treated and untreated samples (*** p value = 0.0005). The WT and the Δ*tasA* data in figure 4 is from the same experiment as the data displayed in this figure and has been used as a control for the comparison between the WT and Δ*tasA* strains and their respective CCCP-treated controls. Source data are provided as a Source Data file.

Supplementary Figure 9

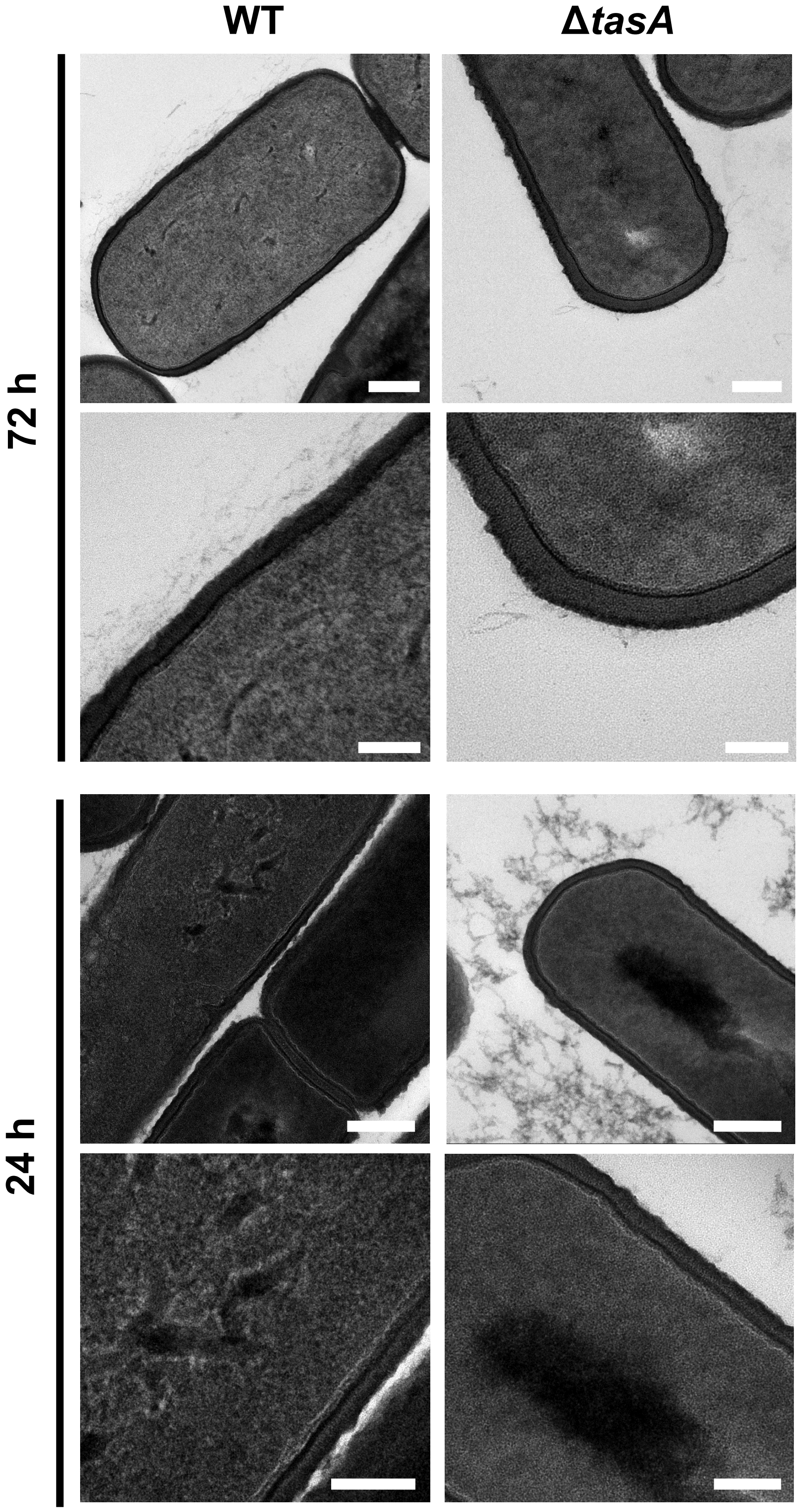

Supplementary Figure 9. No structural alterations were found on the surfaces of *ΔtasA* cells. Transmission electron micrographs of thin sections of embedded WT and *ΔtasA* cells at 24 and 72 h. The details of the cell surfaces show no differences in shape, integrity or thickness. Scale bars = 200 nm (top images) or 100 nm (bottom images). Experiments have been repeated at least three times with similar results

Supplementary Figure 10

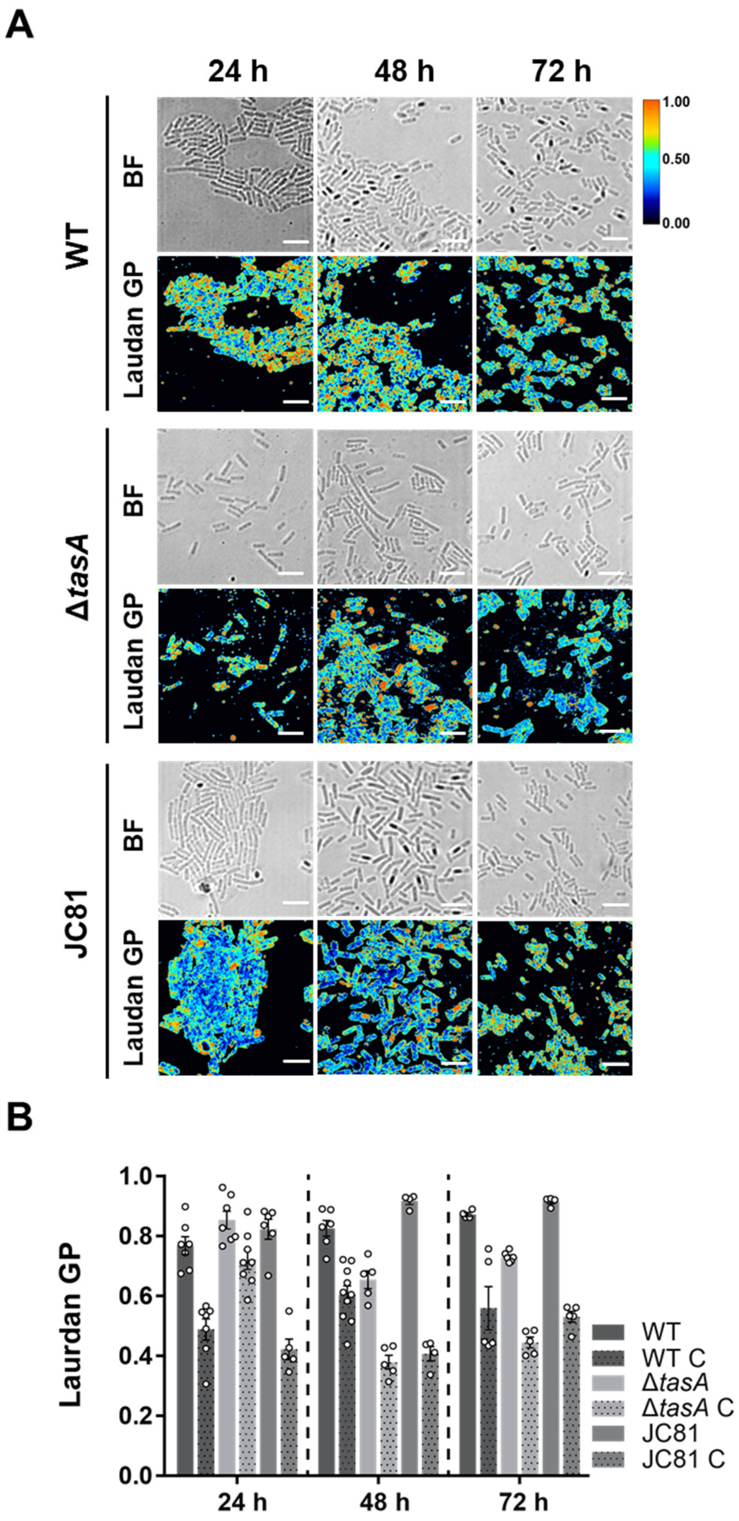

Supplementary Figure 10. Membrane fluidity is increased upon treatment of the cells with benzyl alcohol. A) The Laurdan GP values of WT, *ΔtasA* and JC81 cells treated with 2% benzyl alcohol, a known membrane fluidifier, at 24, 48 and 72 h, were used as controls. The processed Laurdan GP images show decreased Laurdan GP values (i.e., increased membrane fluidity) at all time points in all of the strains upon treatment with benzyl alcohol compared with the values of the untreated samples. A calibration bar (from 0 to 1) is located on the right. Scale bars = 5 µm. B) Quantification of the Laurdan GP values in benzyl alcohol-treated samples. N ranges from 4 to 10 colonies (depending on the sample) examined over three independent experiments. For each experiment and sample, at least three fields-of-view were measured. Average values are shown, with error bars indicating the SEM. The WT and Δ*tasA* data in figure 6 and the JC81 data repeated in figure S15 are from the same experiment as the data displayed in this figure and have been used as a control for the comparison between the untreated samples and the benzyl alcohol treated controls. Source data are provided as a Source Data file.

Supplementary Figure 11

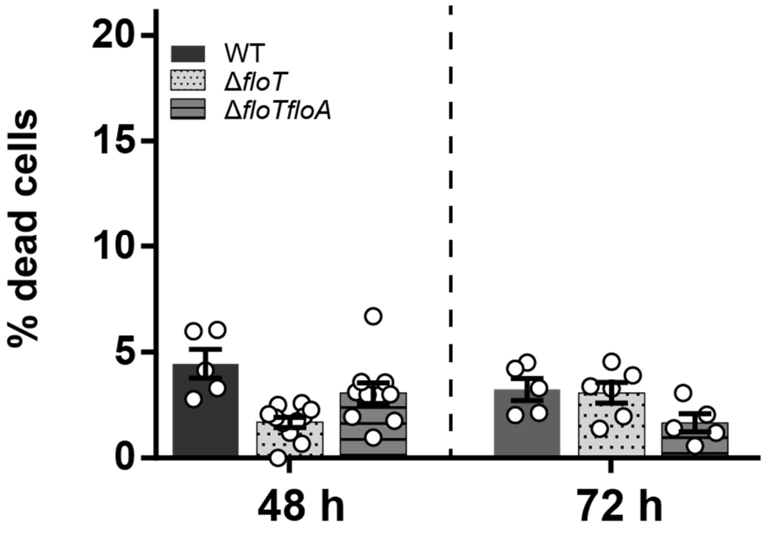

Supplementary Figure 11. Analysis of cell death in flotillin mutants. Quantification by CLSM of the proportion of dead cells found in the Δ*floT* or Δ*floTfloA* colonies grown in solid MSgg for 48 (WT N = 5, Δ*floT* N = 10, Δ*floTfloA* N = 10) or 72 h (WT N = 5, Δ*floT* N = 6, Δ*floTfloA* N = 5). N represents the number of colonies examined over three independent experiments. Average values are shown with error bars representing the SEM. The WT data in figure 4 is from the same experiment as the data displayed in this figure and has been used as a control for the comparison between the WT and the flotillin mutant colonies. Source data are provided as a Source Data file.

Supplementary Figure 12

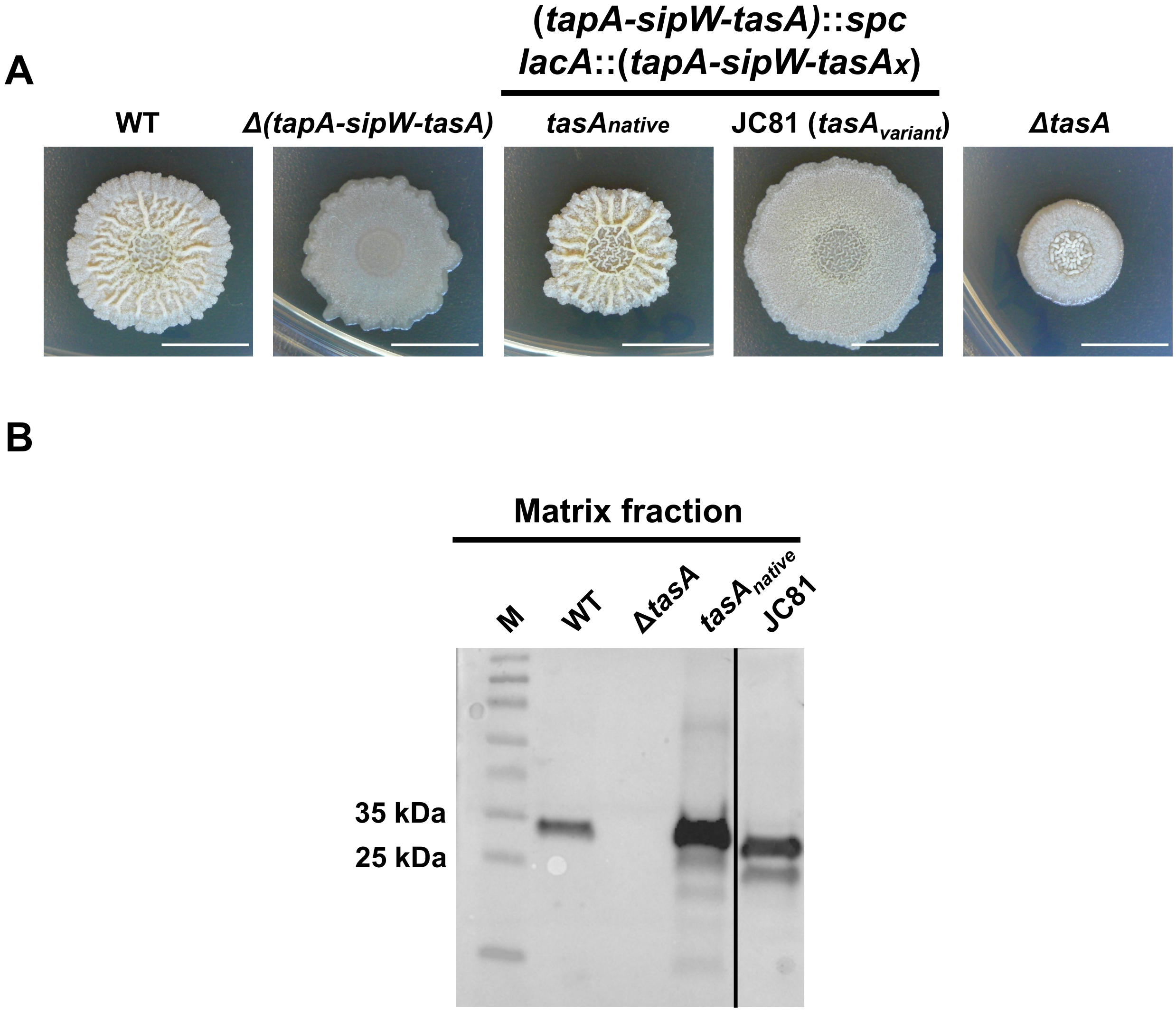

Supplementary Figure 12. Phenotypes of the JC81 strain, which carries an allelic variant of *tasA* (Lys68Ala, Asp69Ala). A) A *Δ*(*tapA-sipW-tasA*) strain was used as the genetic background for introducing the entire operon (in *lacA*, a neutral locus) with different versions of *tasA* in which some amino acids were substituted with alanine via site-directed mutagenesis. Colony morphologies of the different *B. subtilis* mutants on MSgg agar plates. From left to right: WT, *Δ*(*tapA-sipW-tasA*), *Δ*(*tapA-sipW-tasA*)::(*tapA-sipW-tasA*$_{native}$) (containing a WT version of the gene), *Δ*(*tapA-sipW-tasA*)::(*tapA-sipW-tasA*$_{variant}$) (strain JC81, containing the Lys68Ala, Asp69Ala allele) and Δ*tasA*. Scale bars = 1 cm. B) Western blot of the biofilm matrix fraction analyzed with anti-TasA antibody. Experiments have been repeated at least three times with similar results. Immunoblot images have been cropped and spliced for illustrative purposes. Black lines over the blot images delineate boundaries of immunoblot splicing. The two slices shown in the image are derived from a single blot. Raw image of the blot presented in this figure can be found in the source data file available with this manuscript. Source data are provided as a Source Data file.

Supplementary Figure 13

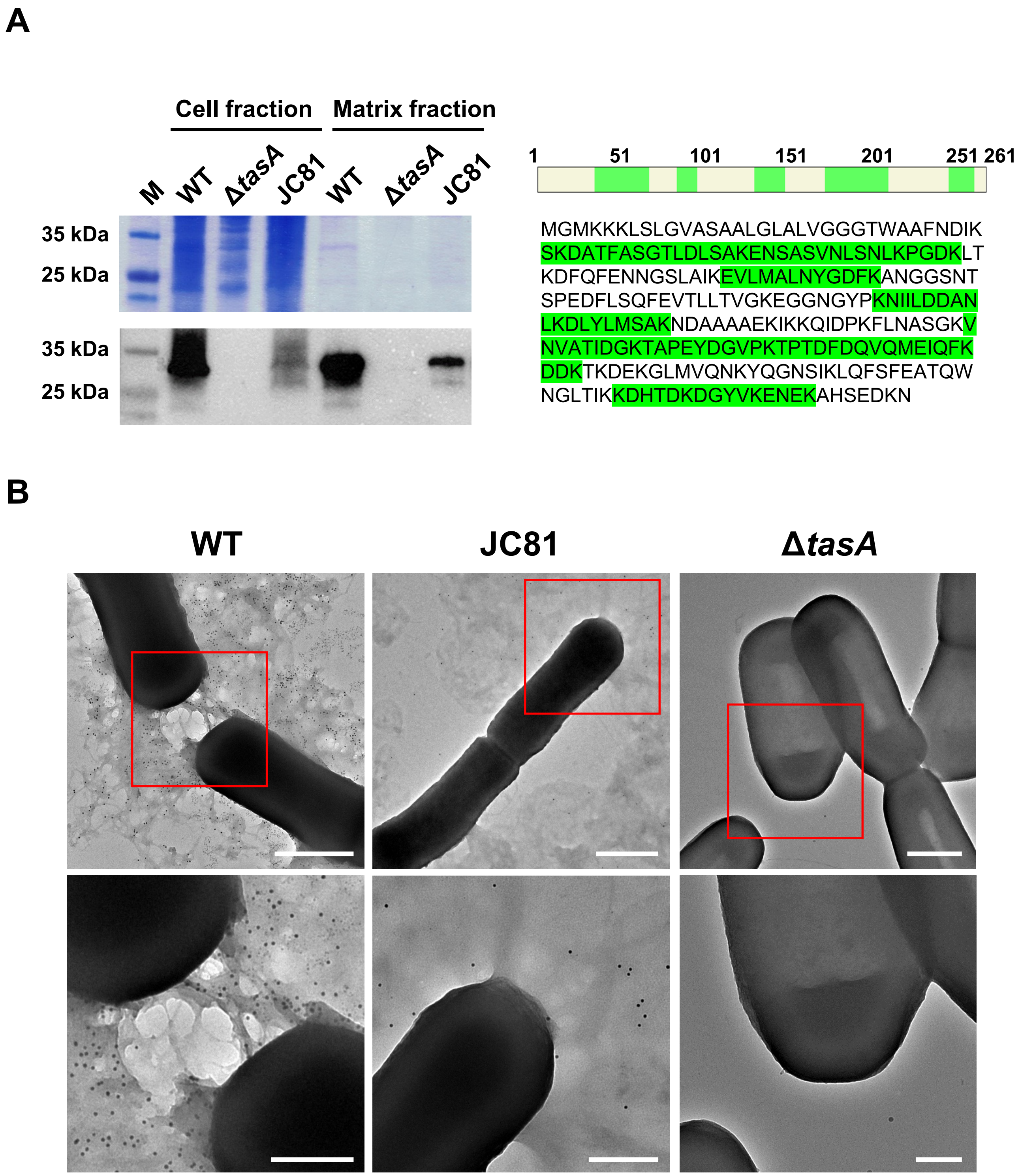

Supplementary Figure 13. The TasA produced by JC81 strain (expressing the TasA variant Lys68Ala, Asp69Ala) corresponds to the mature form of the protein. A) Biofilm fractionation of WT, Δ*tasA* or JC81 colonies and SDS-PAGE of the different fractions followed by Coomassie Blue staining or western blot. The highest band in lane 6 was cut and analyzed via mass spectrometry analysis. The coverage obtained is shown on the right. Experiments have been repeated at least three times with similar results. Gel and blot images have been cropped for illustrative purposes. Raw images can be found in the source data file available with this manuscript. B) Transmission electron micrographs of negatively stained, anti-TasA immunogold labeled samples from cells grown for 48 h in biofilm-inducing conditions. Scale bars = 500 nm (top) and 200 nm (bottom). Experiments have been repeated at least three times with similar results. Source data are provided as a Source Data file.

Supplementary Figure 14

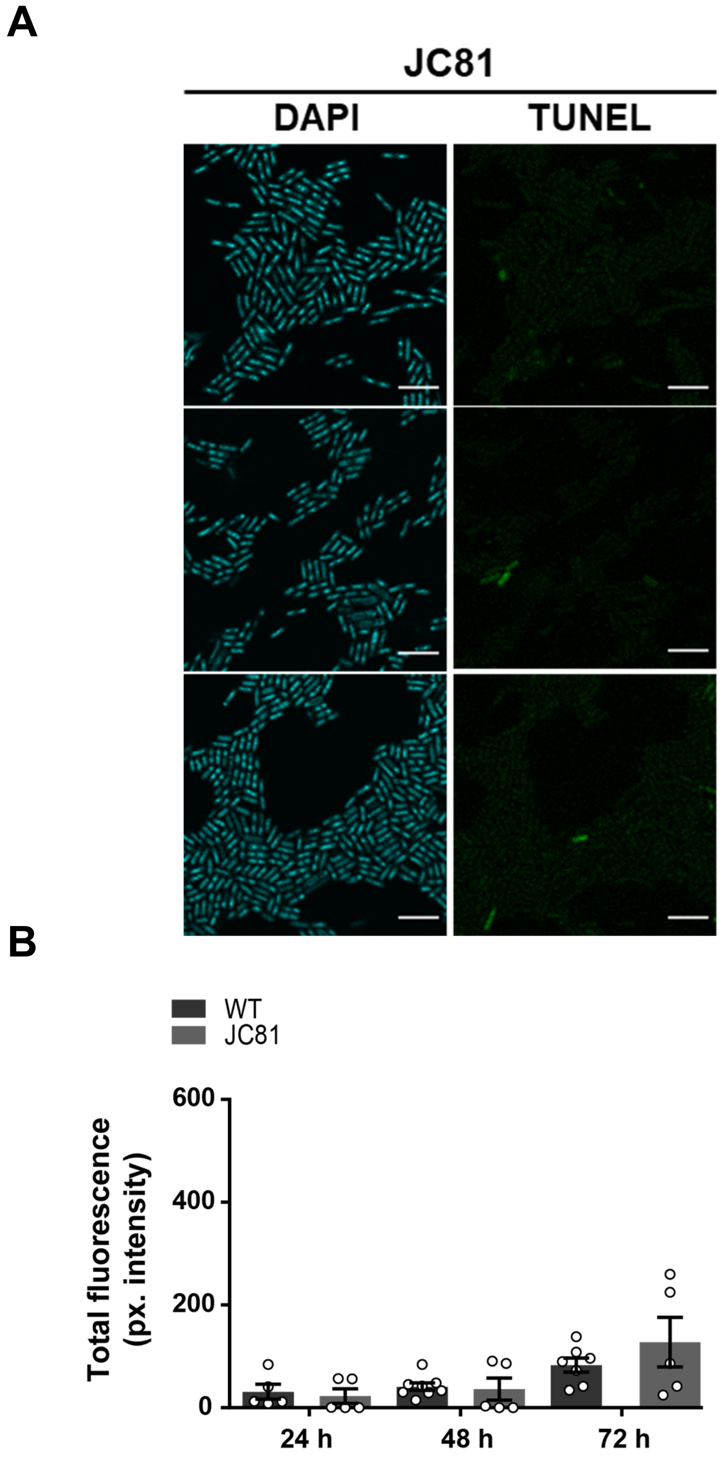

Supplementary Figure 14. JC81 showed no difference in the levels of DNA damage. A) TUNEL assay and CLSM analysis revealed no difference in the levels of DNA damage between JC81 colonies and WT colonies (see figure 5). Scale bars = 5 µm. B) Quantification of the TUNEL signals in WT and JC81 colonies showed similar values. At 24 h N = 5 for both strains. At 48 h N = 8 for the WT strain and N = 5 for Δ*tasA* strain. At 72 h N = 7 for the WT strain and N = 5 for the Δ*tasA* strain. N represents the number of colonies examined over three independent experiments. Average values are shown. For each experiment, at least three fields-of-view were measured. Error bars indicate the SEM. The WT data in figure 5 is from the same experiment as the data displayed in this figure and has been used as a control for the comparison between the WT strain and the JC81 strain. Source data are provided as a Source Data file.

Supplementary Figure 15

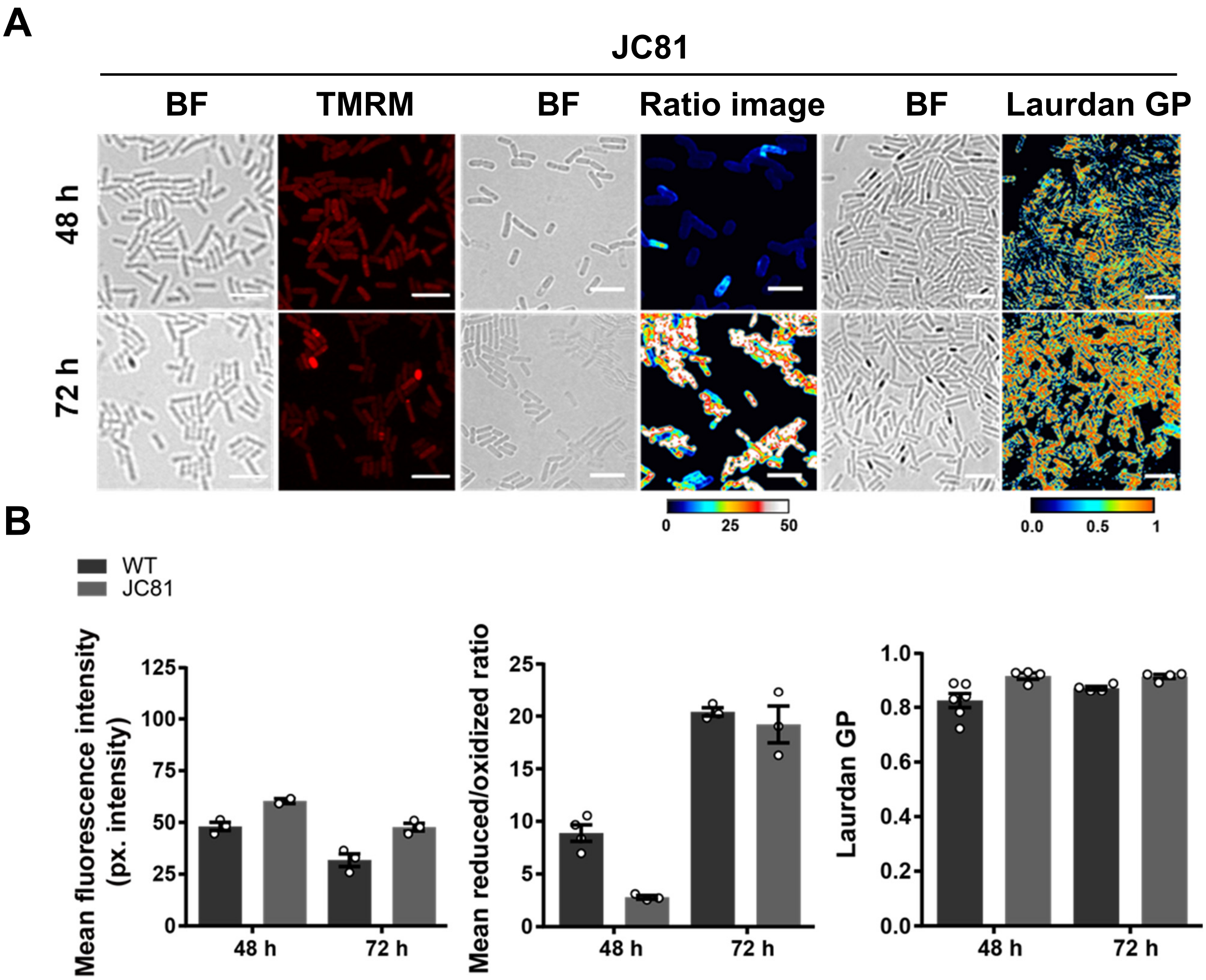

Supplementary Figure 15. The allelic variant of *tasA* (Lys68Ala, Asp69Ala) present in JC81 strain restores the physiological status of Δ*tasA* cells. A) Left panel. A TMRM assay of JC81 cells shows similar membrane potential as WT cells (see figure 6). Center panel. Assessment of the lipid peroxidation levels using BODIPY 581/591 C11 reagent in JC81 cells after treatment with 5mM CuHpx and analysis by CLSM. The ratio images represent the ratio between the two states of the lipid peroxidation sensor: reduced channel(590-613 nm emission)/oxidized channel(509-561nm emission). The ratio images were pseudo-colored depending on the reduced/oxidized ratio values. A calibration bar (from 0 to 50) is located at the bottom of the panel. Confocal microscopy images show that CuHpx treatment was ineffective in the JC81 strain at 72 h. Right panel. Laurdan GP analyzed via fluorescence microscopy. The images were taken at two different emission wavelengths (gel phase, 432 to 482 nm and liquid phase, 509 to 547 nm) that correspond to the two possible states of the Laurdan reagent depending on the lipid environment. The Laurdan GP images represent the Laurdan GP value of each pixel (see Materials and methods). The Laurdan GP images were pseudo-colored depending on the laurdan GP values. A calibration bar (from 0 to 1) is located at the bottom of the set. The Laurdan GP images of JC81 cells show similar membrane fluidity as WT cells at 48 and 72 h. All scale bars are equal to 5 µm. B) Quantification of the TMRM signal (N = 3 colonies examined over three independent experiments), lipid peroxidation (N = 3 colonies examined over three independent experiments, except WT at 48 h, N = 4) and laurdan GP (N = 4 colonies examined over three independent experiments, except WT at 48 h, N = 6) revealed no differences between the WT and JC81 colonies at 48 and 72 h. Average values are shown with error bars representing the SEM. For each experiment and sample, at least three fields-of-view were measured. The WT data in figure 6 is from the same experiment as the data displayed in this figure and has been used as a control for the comparison between the WT strain and the JC81 strain. Source data are provided as a Source Data file.

Supplementary Figure 16

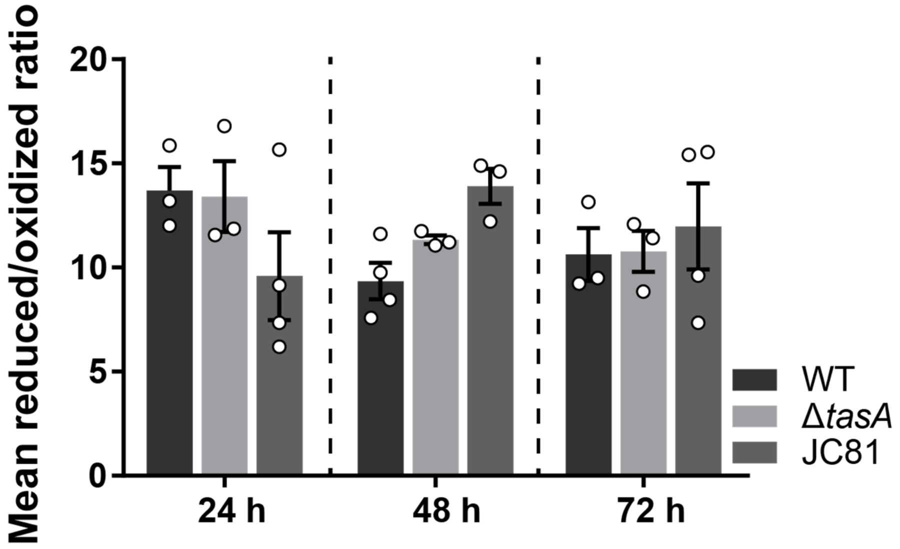

Supplementary Figure 16. Untreated cells from the WT, Δ*tasA* and JC81 strains show no differences in the endogenous levels of lipid peroxidation. Assessment of lipid peroxidation in untreated WT (24 h N = 3, 48 h N = 4 and 72 h N = 3), Δ*tasA* (N = 3 at all times) and JC81 (24 h N = 4, 48 h N = 3 and 72 h N = 4) colonies. N indicates de number of colonies examined over three independent experiments. Quantification of the lipid peroxidation from the ratio images shows no differences in the reduced/oxidized ratio between the strains at the different time points. Average values are shown. Error bars indicate the SEM. Source data are provided as a Source Data file.

Supplementary Figure 17

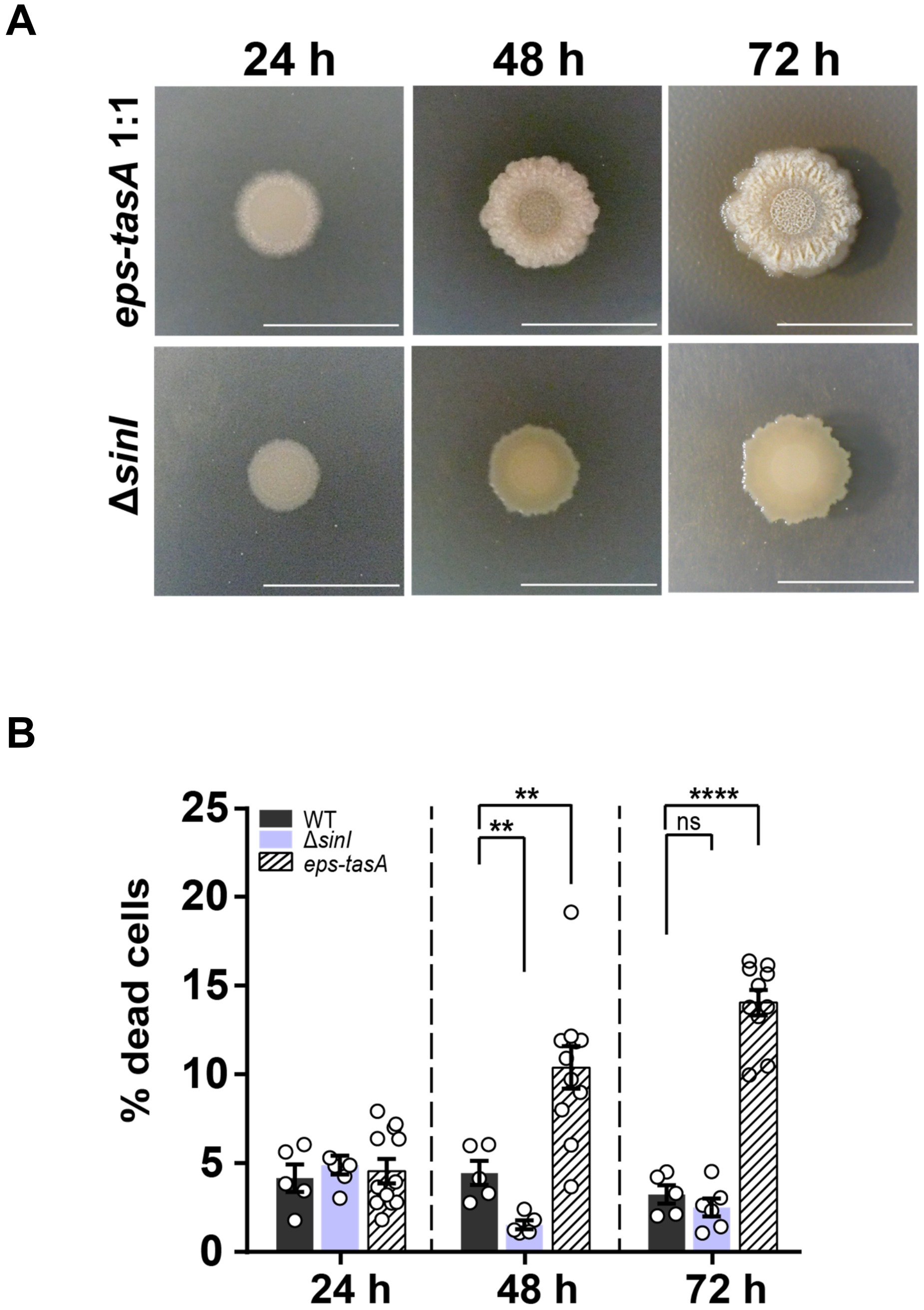

Supplementary Figure 17. The physiological function of TasA does not rely on ECM assembly. A) Colony morphologies at 72 h of a mixture of Δ*tasA* and Δ*eps* strains co-inoculated at a 1:1 ratio and a Δ*sinI* strain. Scale bars = 1 cm. B) Quantification via CLSM of the proportion of dead cells found in the WT (N = 5 at all times), Δ*tasA*-Δ*eps* mixture (24 h N = 11, 48 h N = 9, 72 h N = 10) and Δ*sinI* (N = 5 at all times except at 72 h, where N = 6) colonies grown on solid MSgg for 24, 48, and 72 h. N represents the number of colonies examined over three independent experiments. Average values are shown with error bars representing the SEM. Statistical significance was assessed via two-tailed independent t-tests at each time-point comparing with the WT strain (at 48 h, ** p value = 0.0067 for *eps-tasA* and ** p value = 0.0037 for Δ*sinI*, at 72 h, **** p value < 0.0001). The WT data in figure 4 is from the same experiment as the data displayed in this figure and has been used as a control for the comparison between the WT, Δ*tasA*-Δ*eps* mixture and Δ*sinI* colonies. Source data are provided as a Source Data file.

Supplementary Figure 18

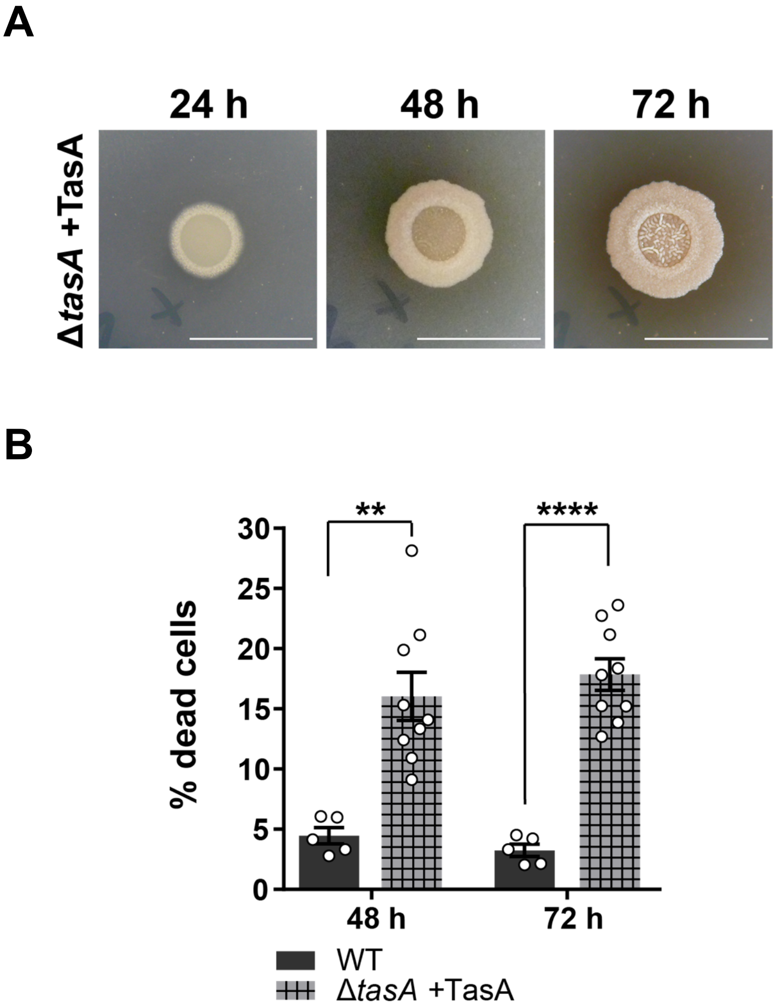

Supplementary Figure 18. Exogenous addition of TasA to solid medium does not complement the functionality of TasA. A) Colony morphologies of a Δ*tasA* strain complemented with 80 µg of purified TasA protein on solid medium at 24, 48 and 72 h. Scale bars = 1 cm. B) Quantification via CLSM of the proportions of dead cells found in the WT (N = 5) and complemented Δ*tasA* colonies (N = 8 at 48 h and N = 9 at 72 h) grown in solid MSgg for 48, and 72 h. N represents the number of colonies examined over three independent experiments. Average values are shown with error bars representing the SEM. Statistical significance was assessed via two-tailed independent t-tests at each time-point comparing with the WT strain (**** $p<0.0001$, ** p value = 0.0013). The WT data in figure 4 is from the same experiment as the data displayed in this figure and has been used as a control. Source data are provided as a Source Data file.

Supplementary Figure 19

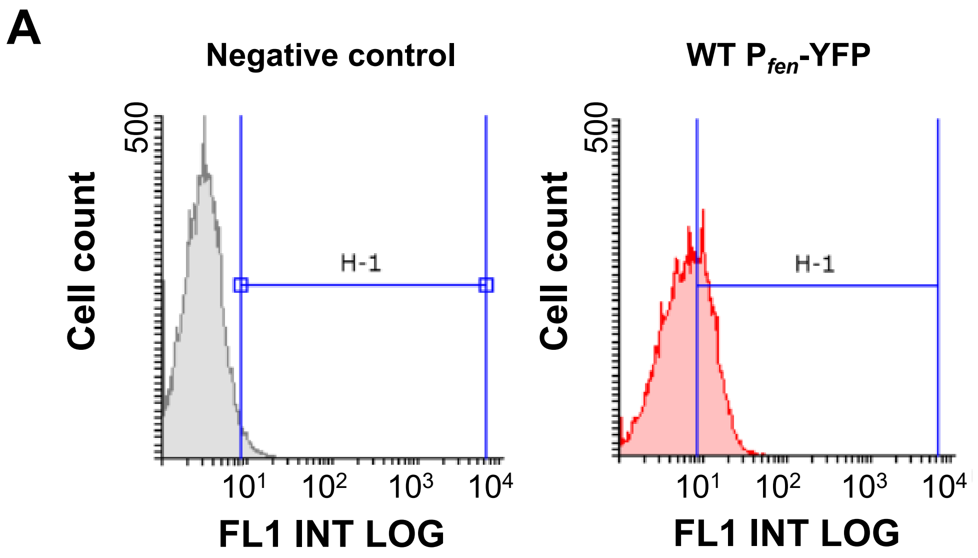

B

	Events	% of Vis.	Mean
All events	30000	100	8.42420481
H-1	10860	36.2	14.18917392

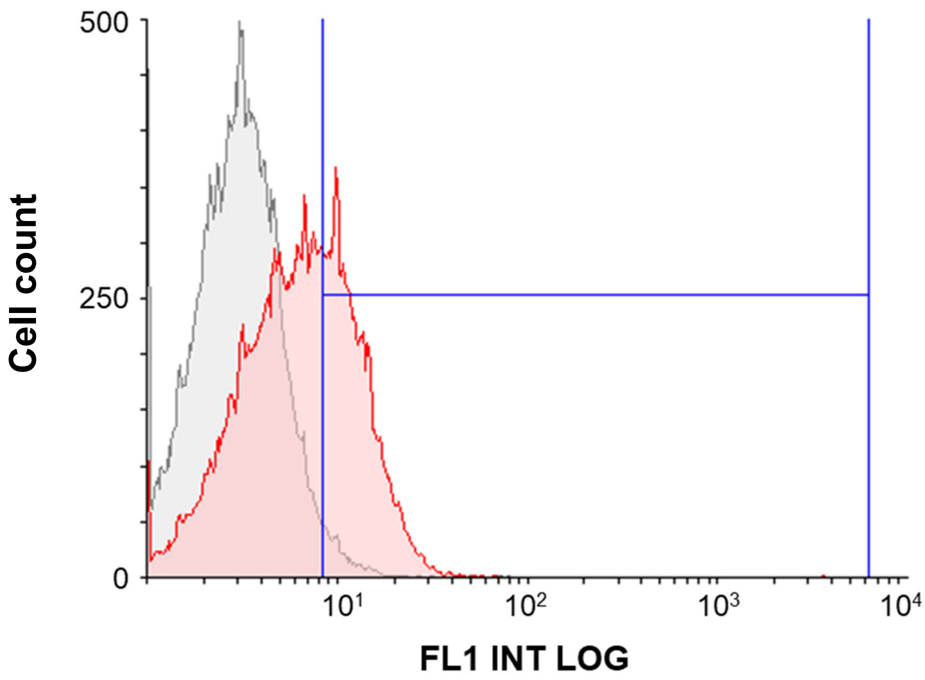

Supplementary Figure 19. Gating of flow cytometry data. A) The fluorescence negative control is used to define the positive population in a labelled sample (region H1) (e. g. the WT strain at 72 h bearing a transcriptional fusion of the fengycin operon promoter to YFP). B) An overlay histogram is created from these data, and the % of positive cells is calculated based on the negative controls. The flow cytometry data shown in figure 3A is from the same experiment as the data shown in this figure and are repeated here for illustrative purposes.

Supplementary Table 1. Flow cytometry – Quadrant statistics.

Time	Sample	HPF (Quad. 1)	HPF + CTC (Quad. 2)	Unstained (Quad. 3)	CTC (Quad. 4)
**24h**	WT	10.79 ± 2.22	17.83 ± 1.83	20.12 ± 1.01	51.27 ± 1.98
	Δ*tasA*	27.70 ± 0.79	14.68 ± 5.94	29.24 ± 8.62	28.40 ± 3.24
	JC81	4.16 ± 1.68	15.58 ± 0.04	20.68 ± 8.00	59.59 ± 9.64
**48h**	WT	0.45 ± 0.16	2.06 ± 0.48	22.38 ± 3.24	75.12 ± 2.92
	Δ*tasA*	2.72 ± 2.44	4.84 ± 3.62	23.51 ± 7.53	68.93 ± 13.58
	JC81	3.39 ± 0.68	11.24 ± 0.67	21.97 ± 4.91	70.64 ± 5.20
**72h**	WT	8.44 ± 6.43	51.10 ± 9.51	6.02 ± 4.77	34.44 ± 1.69
	Δ*tasA*	1.40 ± 0.85	4.65 ± 0.80	33.43 ± 12.24	60.46 ± 12.89
	JC81	1.53 ± 0.34	3.98 ± 0.54	26.25 ± 2.16	68.25 ± 3.03

Supplementary Table 2. Bacterial strains used in this study

Bacterial strain	Genotype	Source
Bacillus subtilis 168	Prototroph	Laboratory collection
Bacillus subtilis NCIB3610	Wild type. Undomesticated strain	Laboratory collection
CA017	*Bacillus subtilis* NCIB3610 *tasA::km*	(Vlamakis *et al*., 2008) (23)
FC268	*Bacillus subtilis* NCIB3610<br>*(tapA-sipW-tasA)::spc,*<br>*amyE::(tapA(13-234)-sipW-tasA) (cm)*	(Chu *et al*., 2006) (9)
SSB488	*Bacillus subtilis* NCIB3610<br>*epsA-O::tet*	(Branda *et al*., 2006) (101)
SSB149	*Bacillus subtilis* NCIB3610<br>*(tapA-sipW-tasA)::spc*	(Branda *et al*., 2006) (101)
PSK0156	*Bacillus subtilis* 168<br>*ppsB* ΩTn*10 (spc)*	(Straight *et al*., 2007) (128)
DL1207	*Bacillus subtilis* NCIB 3610<br>*amyE::floT-yfp (spc)*	(López and Kolter, 2010) (73)
DL811	*Bacillus subtilis* NCIB 3610<br>*sinI::spc*<br>*lacA::*P_{srfAA}*-lacZ*	Kindly provided by Dr. Daniel López
DL1419	*Bacillus subtilis* NCIB 3610<br>*floT::km*<br>*floA::mls*	(Yepes *et al*. 2012) (71)
DRBB5	*Bacillus subtilis* NCIB 3610<br>*tasA::km* (*km*)<br>*amyE::floT-yfp (spc)*	This study
JC97	*Bacillus subtilis* NCIB3610<br>*bslA::spc*	This study
YNG001	*Bacillus subtilis* NCIB3610<br>*amyE::*P_{pps}*-yfp (spc)*	This study
YNG002	*Bacillus subtilis* NCIB3610<br>*tasA::km*<br>*amyE::*P_{pps}*-yfp (spc)*	This study
YNG003	*Bacillus subtilis* NCIB3610<br>*(tapA-sipW-tasA)::spc*<br>*lacA::(tapA-sipW-tasA*$_{(Lys68Ala, Asp69Ala)}$*) (mls)*<br>*amyE::*P_{pps}*-yfp (cm)*	This study
YNG004	*Bacillus subtilis* NCIB3610<br>*ppsB* ΩTn*10 (spc)*	This study
JC81	*Bacillus subtilis* NCIB3610<br>*(tapA-sipW-tasA)::spc*<br>*lacA::(tapA-sipW-tasA*$_{(Lys68Ala, Asp69Ala)}$*) (mls)*	This study
JC149	*Bacillus subtilis* NCIB3610<br>*(tapA-sipW-tasA)::spc*<br>*lacA::(tapA-sipW-tasA*$_{(Lys4Ala, Lys5Ala, Lys6Ala)}$ *(mls)*	This study
JC70	*Bacillus subtilis* NCIB3610<br>*(tapA-sipW-tasA)::spc*<br>*lacA::(tapA-sipW-tasA*$_{native}$*) (mls)*	This study
JC138	*Bacillus subtilis* NCIB3610<br>*floT::km*	This study
JC189	*Bacillus subtilis* NCIB3610<br>*sinI::spc*	This study
DRBB2	*Bacillus subtilis* NCIB3610<br>*lacA::(tapA-sipW-tasA-mCherry) (mls)*<br>*amyE::floT-yfp (spc)*	This study

Supplementary Table 3. Primers used in this study.

Name	Sequence (5' – 3')	Purpose
Ppps-ecoRI.F	AAAAAAGAATTCTCACTTTATATCCGGAAATTTGA	Cloning P_{pps} into pKM003
Ppps-HindIII.R	AAAAAAAAGCTTAACGGATTCCCTCCAGTTCT	Cloning P_{pps} into pKM003
KD_AA_68-70	GATCCGTTATTTTCAAATTGGAAAGCCGCTGTCAACTTAT CTCCCGGCTTTAG	Site-directed mutagenesis of residues 68 and 69 in TasA aminoacid sequence
KD_AA_68-70_as	CTAAAGCCGGGAGATAAGTTGACAGCGGCTTTCCAATTT GAAAATAACGGATC	Site-directed mutagenesis of residues 68 and 69 in TasA aminoacid sequence
KKK4-5-6AAA	TCAATAAAAGGGGAGCTTACCATGGGTATGGCAGCGGC ATTGAGTTTAGGAGTTGCTTCTGCAGCACT	Site-directed mutagenesis of residues 4, 5 and 6 in TasA aminoacid sequence
KKK4-5-6AAA_as	AGTGCTGCAGAAGCAACTCCTAAACTCAATGCCGCTGC CATACCCATGGTAAGCTCCCCTTTTATTGA	Site-directed mutagenesis of residues 4, 5 and 6 in TasA aminoacid sequence
TasA_1_mutb	AAAAAGTCGACATTAGATAGTGAATGGGAGAAATTGG	Cloning of (*tapA-sipW-tasA*) into pDR183
YSRI_2	AAAAAGGATCCGCTATAAGGATCAAATGAAATCG	Cloning of (*tapA-sipW-tasA*) into pDR183
bslAUP-Fw	TTCGAGCTCGGTACCCGGGGATCCTTAAAGACTTTGATT GTCGTCAG	Construction of *bslA* mutant.
bslAUP-Rv	CGTTACGTTATTAAACAAAATTCCCCCTAAAAAATG	Construction of *bslA* mutant.
Spc-Fw	GGGGAATTTTGTTTAATAACGTAACGTGACTGG	Construction of *bslA* mutant.
Spc-Rv	ATCCGGCTTGTACGCAAGGGTTTATTGTTTTC	Construction of *bslA* mutant.
bslaDOWN-Fw	AATAAACCCTTGCGTACAAGCCGGATGGATAAAATG	Construction of *bslA* mutant.
bslADOWN-Rv	AAGCTTGCATGCCTGCAGGTCGACTTGTAAAAGACCGG TTAACGC	Construction of *bslA* mutant.
amyEUP-Fw	AGTGAATTCGAGCTCGGTACCCGGGGCCGTTTACCGTT CGCCATAAG	Construction of strain YNG004
amyEUP-Rv	GGATATAAAGTGATCTTGACACTCCTTATTTGATTTTTTG AAG	Construction of strain YNG004
Ppps-Fw	AGGAGTGTCAAGATCACTTTATATCCGGAAATTTG	Construction of strain YNG004
Ppps-Rv	TGACGAAGCATGGAACGGATTCCCTCCAGTTC	Construction of strain YNG004
Yfp-Fw	GAGGGAATCCGTTCCATGCTTCGTCAATGTATATG	Construction of strain YNG004
Yfp-Rv	AATATATATTTTATTATTTGTATAGTTCATCCATGC	Construction of strain YNG004
Cat-Fw	ACTATACAAATAATAAAATATATATTTATGTTACAGTAATA TTGAC	Construction of strain YNG004
Cat-Rv	TGCCGGCATTTCGTTATAAAAGCCAGTCATTAGG	Construction of strain YNG004
amyEDOWN-Fw	CTGGCTTTTATAACGAAATGCCGGCAATGCTG	Construction of strain YNG004
amyEDOWN-Rv	GCATGCCTGCAGGTCGACTCTAGAGGGGCAAGGCTAGA CGGGAC	Construction of strain YNG004

Supplementary Table 4. Primers specifically designed for qRT-PCR assays

Primers pairs	Primers	Sequence (5'-3')	Gene	Amplicon (bp)
1	fenD-for<br>fenD-rev	ATGATGGACGGTTGGTGC<br>CTGCTCTTTGTCTTGCTC	*fenD*	147
2	alsS-for<br>alsS-rev	AGTGGTTTCTGTCTCTGGTG<br>AATTGGTGCTTTTAGTCG	*alsS*	82
4	albE-for<br>albE-rev	ACTTGTCTTTCCCTTCTTCTC<br>CGCCTTTATCCTGTCTCTCC	*albE*	185
5	bacB-for<br>bacB-rev	GCAAGAAACGACACAGACCA<br>CTACCCAATCCTCAACAAAGAAC	*bacB*	160
6	srfAA-for<br>srfAA-rev	TGGTGATGCTTTCCGCTTAC<br>GAGTGATTTCTGCCCGCT	*srfAA*	94
7	rpsJ-for<br>rpsJ-rev	TCTGGTCCGATTCCGTTGCC<br>CAGTTTGTGGTGTTGGGTTCA	*rpsJ*	142
