# Supplementary material for: Dual functionality of the amyloid protein TasA in Bacillus physiology and fitness on the phylloplane
Source: Nat Commun. 2020 Apr 20;11:1859. doi: 10.1038/s41467-020-15758-z (PMC7171179; doi:10.1038/s41467-020-15758-z)
Supplement: Supplementary file 3 — Description of Additional Supplementary Files [file 41467_2020_15758_MOESM3_ESM.pdf]

Description of Additional Supplementary Files

File name: Supplementary Data 1

Description: Differentially expressed genes in the Δ*tasA* strain compared to the WT strain at 24 h. Genes with a Log2 FC value >1 or <-1 were considered as differentially expressed. Only genes that showed a combined p value <0.05 were considered.

File name: Supplementary Data 2

Description: Differentially expressed genes in the Δ*tasA* strain compared to the WT strain at 48 h. Genes with a Log2 FC value >1 or <-1 were considered as differentially expressed. Only genes that showed a combined p value <0.05 were considered.

File name: Supplementary Data 3

Description: Differentially expressed genes in the Δ*tasA* strain compared to the WT strain at 72 h. Genes with a Log2 FC value >1 or <-1 were considered as differentially expressed. Only genes that showed a combined p value <0.05 were considered.
