# Supplementary material for: Dual functionality of the amyloid protein TasA in Bacillus physiology and fitness on the phylloplane
Source: Nat Commun. 2020 Apr 20;11:1859. doi: 10.1038/s41467-020-15758-z (PMC7171179; doi:10.1038/s41467-020-15758-z)
Supplement: Supplementary file 7 — Reporting Summary [file 41467_2020_15758_MOESM7_ESM.pdf]

nature research

Corresponding author(s): Diego Romero

Last updated by author(s): 19/03/2020

Reporting Summary

Nature Research wishes to improve the reproducibility of the work that we publish. This form provides structure for consistency and transparency in reporting. For further information on Nature Research policies, see Authors & Referees and the Editorial Policy Checklist.

Statistics

For all statistical analyses, confirm that the following items are present in the figure legend, table legend, main text, or Methods section.

n/a	Confirmed	
☐	☒	The exact sample size (n) for each experimental group/condition, given as a discrete number and unit of measurement
☐	☒	A statement on whether measurements were taken from distinct samples or whether the same sample was measured repeatedly
☐	☒	The statistical test(s) used AND whether they are one- or two-sided<br>*Only common tests should be described solely by name; describe more complex techniques in the Methods section.*
☒	☐	A description of all covariates tested
☐	☒	A description of any assumptions or corrections, such as tests of normality and adjustment for multiple comparisons
☐	☒	A full description of the statistical parameters including central tendency (e.g. means) or other basic estimates (e.g. regression coefficient) AND variation (e.g. standard deviation) or associated estimates of uncertainty (e.g. confidence intervals)
☐	☒	For null hypothesis testing, the test statistic (e.g. F, t, r) with confidence intervals, effect sizes, degrees of freedom and P value noted<br>*Give P values as exact values whenever suitable.*
☒	☐	For Bayesian analysis, information on the choice of priors and Markov chain Monte Carlo settings
☒	☐	For hierarchical and complex designs, identification of the appropriate level for tests and full reporting of outcomes
☒	☐	Estimates of effect sizes (e.g. Cohen's d, Pearson's r), indicating how they were calculated

Our web collection on statistics for biologists contains articles on many of the points above.

Software and code

Policy information about availability of computer code

Data collection	RNA-seq data were collected using NextSeq System Suite v2.2.0. Confocal microscopy images were taken using Leica Application Suite Advance Fluorescence v2.7.3.9723. Flow cytometry data were collected using Gallios™ Software v1.2. Electron microscopy images were taken using TIA FEI Imaging Software v4.14. MALDI-TOF/TOF data were collected using the Bruker Daltonics FlexControl software v3.4. HPLC-ESI-MS/MS data corresponding to the identification of proteins in gel cut bands was acquired using ProteinScape 3 software from Bruker coupled to Mascot v3.1 (Matrix Science).
Data analysis	The raw reads were pre-processed with SeqTrimNext v2.0.60 using the specific NGS technology configuration parameters. This pre-processing removes low quality, ambiguous and low complexity stretches, linkers, adaptors, vector fragments, and contaminated sequences while keeping the longest informative parts of the reads. SeqTrimNext also discarded sequences below 25 bp. Subsequently, clean reads were aligned and annotated using the Bacillus subtilis subsp. subtilis str. 168 genome (NC_000964.3) as the reference with Bowtie v2.2.9 in BAM files, which were then sorted and indexed using SAMtools v1.484110. Uniquely localized reads were used to calculate the read number value for each gene via Sam2counts v20131126 (https://github.com/vsbuffalo/sam2counts) . Differentially expressed genes (DEGs) between WT and ΔtasA were analyzed via DEgenes Hunter v1.0, which provides a combined P-value from edgeR and DEseq2 v1.26.0 that allowed differentially expressed gene ranking. For each gene, P-value < 0.05 and log2-fold change > 1 or < -1 were considered as the significance threshold. Heatmap and DEGs clusterization was performed using ComplexHeatmap v2.2.0 in Rstudio Desktop v1.2.5033. STEM v1.3.12 was used to model temporal expression profiles independent of the data. Only profiles with a pvalue > 0.05 were considered in this study. The DEGs annotated with the Bacillus subtilis subsp. subtilis str. 168 genome were used to identify the Gene Ontology functional categories using sma3s v2.2 and TopGo Software v2.38.1. Gephi software v0.9.2 (https://gephi.org) was used to generate the DEG networks, and the regulon list was downloaded from subtiwiki (http://subtiwiki.uni-goettingen.de).<br><br>Flow cytometry data was analyzed using Gallios™ Software v1.2 and further analyzed using Kaluza Analysis v1.3 and Flowing Software v2.5.1.<br><br>For the MALDI-TOF/TOF data the mass spectra were processed using Bruker Daltonics FlexAnalysis v3.4.

For protein identification from HPLC-ESI-MS/MS data, the software ProteinScape 3 (Bruker Daltonics) coupled to the search engine Mascot 3.1 (Matrix Science) was used, matching the MS/MS data against the Swiss-Prot and NCBInr databases.

For CLSM and fluorescence microscopy data analysis, image processing was performed using Leica Application Suite X v3.0.6.20104, FIJI/ImageJ v2.0.0-rc-69/1.52p and Imaris version v7.6 (Bitplane) softwares.

For manuscripts utilizing custom algorithms or software that are central to the research but not yet described in published literature, software must be made available to editors/reviewers. We strongly encourage code deposition in a community repository (e.g. GitHub). See the Nature Research guidelines for submitting code & software for further information.

Data

Policy information about availability of data

All manuscripts must include a data availability statement. This statement should provide the following information, where applicable:

- Accession codes, unique identifiers, or web links for publicly available datasets
- A list of figures that have associated raw data
- A description of any restrictions on data availability

The RNA-seq data that support the findings of this study have been deposited in GEO database with the accession code GSE124307.

Field-specific reporting

Please select the one below that is the best fit for your research. If you are not sure, read the appropriate sections before making your selection.

☒ Life sciences ☐ Behavioural & social sciences ☐ Ecological, evolutionary & environmental sciences

For a reference copy of the document with all sections, see nature.com/documents/nr-reporting-summary-flat.pdf

Life sciences study design

All studies must disclose on these points even when the disclosure is negative.

Sample size	Sample-size calculation was not initially performed but a sufficient number of replicates were taken based on literature standards, prior experience with these types of data, and analysis of the current data, which indicates the reproducibility of the data. The number of replicates is sufficient in order to get statistically confident results, as demonstrated in the manuscript. In all the experiments performed, standard deviation and standard error were calculated and the corresponding statistical tests were performed when needed.
Data exclusions	No data were excluded from the study.
Replication	At least three replicates were taken in all the experiments performed. In all the cases the experiments were successfully replicated
Randomization	Randomization is not relevant in this case as our study is mainly focused in the physiology of a bacterial species
Blinding	Blinding is not relevant in this case as our study is mainly focused in the physiology of a bacterial species

Reporting for specific materials, systems and methods

We require information from authors about some types of materials, experimental systems and methods used in many studies. Here, indicate whether each material, system or method listed is relevant to your study. If you are not sure if a list item applies to your research, read the appropriate section before selecting a response.

Materials & experimental systems

n/a	Involved in the study	
☐	☒	Antibodies
☒	☐	Eukaryotic cell lines
☒	☐	Palaeontology
☒	☐	Animals and other organisms
☒	☐	Human research participants
☒	☐	Clinical data

Methods

n/a	Involved in the study	
☒	☐	ChIP-seq
☐	☒	Flow cytometry
☒	☐	MRI-based neuroimaging

Antibodies

Antibodies used	The anti-TasA antibody used for immunodetection studies in this work was kindly provided by professor Adam Driks (Stover and Driks, 1999). The anti-GFP antibody used in this study was commercially available at Clontech (Living Colors® Full-Length GFP Polyclonal Antibody, cat. nos. 632592 & 632593). The secondary antibody used in western blot was commercially available at

Bio-Rad (Goat Anti-Rabbit IgG (H + L)-HRP Conjugate cat. no. 1706515)

Validation

The anti-TasA primary antibody was validated elsewhere in the literature (Stover and Driks, 1999).

The anti-GFP antibody was validated by the supplier as follows: "The quality and performance of this lot of Living Colors Full-Length GFP Polyclonal Antibody was tested by Western blot analysis using lysate made from an HEK 293 cell line stably expressing AcGFP1. After cells were collected and lysed using SDS sample buffer, the lysate (10 µl; equivalent to 35,000 cells) was electrophoresed on a 12% SDSpolyacrylamide gel and transferred to a nitrocellulose membrane. The blot was probed with the Living Colors Full-Length GFP Polyclonal Antibody (diluted 1:1,000), followed by a secondary goat anti-rabbit antibody conjugated to horseradish peroxidase (HRP). The HRP signal was detected by chemiluminescence. A 30 kDa band corresponding to AcGFP1 was observed in the lane loaded with the AcGFP1 cell lysate. A band of this size was not detected in the lysate of untransfected HEK 293 cells." The Goat Anti-Rabbit IgG (H + L)-HRP Conjugate (cat. no. 1706515) secondary antibody from Bio-Rad was validated by the supplier as follows: "Specific for rabbit IgG, heavy and light chain. The cross-reactivities of anti-rabbit IgG antibody are tested in an ELISA. Minimum cross-reactivity to human IgG. 1:2,000-1:5,000 dilution can be used (Coligan, J., 1997)."

Flow Cytometry

Plots

Confirm that:

☒ The axis labels state the marker and fluorochrome used (e.g. CD4-FITC).

☒ The axis scales are clearly visible. Include numbers along axes only for bottom left plot of group (a 'group' is an analysis of identical markers).

☒ All plots are contour plots with outliers or pseudocolor plots.

☒ A numerical value for number of cells or percentage (with statistics) is provided.

Methodology

Sample preparation

Cells were grown on MSgg agar at 30 ºC. At different time points, colonies were recovered in 500 µL of PBS and resuspended with a 25 5/8 G needle. Suspensions were gently sonicated with 4-5 5 seconds pulses at 20% amplitude to ensure complete resuspension, and then if needed, the cells were fixed in 4% paraformaldehyde in PBS and washed three times in PBS. To evaluate the physiological status of the different B. subtilis strains, cells were stained without fixation for 30 minutes with 5 mM 5-cyano-2,3-ditolyltetrazolium chloride (CTC) and 15 µM 3-(p-hydroxyphenyl) fluorescein (HPF). The flow cytometry runs were performed with 200 µl of cell suspensions in 800 µL of GTE buffer (50 mM glucose, 10 mM EDTA, 20 mM Tris-HCl; pH 8).

Instrument

Beckman Coulter Gallios™

Software

Gallios™ Software v1.2

Cell population abundance

Sample acquisition was performed at 1,000 event/s. 20,000 events were collected and stored for each strain sample and experiment.

Gating strategy

Negative controls corresponding to unstained bacterial cells (or unlabelled cells corresponding to each strain for the promoter expression analysis) were used to discriminate the populations of stained bacteria in the relevant experiments and for each dye.

☒ Tick this box to confirm that a figure exemplifying the gating strategy is provided in the Supplementary Information.
